# HIV-Infected Patients as a Model of Aging

**DOI:** 10.1128/spectrum.00532-23

**Published:** 2023-04-24

**Authors:** Boško Toljić, Jelena Milašin, Silvio R. De Luka, Gordana Dragović, Djordje Jevtović, Aleksandar Maslać, Jasna L. Ristić-Djurović, Alexander M. Trbovich

**Affiliations:** a School of Dental Medicine, University of Belgrade, Belgrade, Serbia; b School of Medicine, University of Belgrade, Belgrade, Serbia; c Blood Transfusion Institute of Serbia, Belgrade, Serbia; d Institute of Physics, University of Belgrade, Belgrade, Serbia; Oklahoma State University College of Veterinary Medicine

**Keywords:** HIV, aging, pharmacotherapy, iron, telomere

## Abstract

We appraised the relationship between the biological and the chronological age and estimated the rate of biological aging in HIV-infected patients. Two independent biomarkers, the relative telomere length and iron metabolism parameters, were analyzed in younger (<35) and older (>50) HIV-infected and uninfected patients (control group). In our control group, telomeres of younger patients were significantly longer than telomeres of older ones. However, in HIV-infected participants, the difference in the length of telomeres was lost. By combining the length of telomeres with serum iron, ferritin, and transferrin iron-binding capacity, a new formula for determination of the aging process was developed. The life expectancy of the healthy population was related to their biological age, and HIV-infected patients were biologically older. The effect of antiretroviral HIV drug therapies varied with respect to the biological aging process.

**IMPORTANCE** This article is focused on the dynamics of human aging. Moreover, its interdisciplinary approach is applicable to various systems that are aging.

## INTRODUCTION

The dynamic of aging is mostly unpredictable, and it varies considerably between different individuals, organs, tissues, and even the same cell types (1–3). Two major proposed mechanisms of aging are programmed aging and stochastic aging. Programmed aging is based on a limited proliferative capacity of cells and puts the emphasis on the influence of intrinsic factors on a cell’s life span (4). The stochastic theories of aging include the contribution of external factors to the aging process and are proposed as an alternative for the programmed aging in cases where genetically identical organisms exhibit discordance in the aging rate (5–7). The complex interactions of both intrinsic and extrinsic factors are most likely responsible for distinct aging patterns among individuals. Moreover, these interactions could result in an unmatched rate of biological and chronological age in the same individual. Various biomarkers of aging were proposed as potential tools for addressing this issue (8–10). We compared biological and chronological aging by focusing on the relative telomere length (RTL) and iron metabolism parameters.

Telomeres serve as protective caps at the end of chromosomes, and they progressively shorten with every cell cycle due to the incapacity of DNA replicative machinery to maintain unchanged those outermost parts of the human genome (4). The telomere shortening is different in various cell types due to unequal mitotic activities of cells (11).

The accumulation of iron in cells has been suggested as a biomarker of the aging process (12), although it has not yet been investigated as such. The iron uptake into a body and transfer to the target cells are controlled by several proteins located in enterocytes and the bloodstream (13). Inside cells, iron is used for the synthesis of different biomolecules involved in various activities, or it is stored bound to ferritin. Since iron excretion from a body is not tightly regulated, cell iron deposits increase over time and lead to age-related changes in cell metabolism (14).

Understanding biological aging is of enormous importance for the individuals infected with human immunodeficiency virus (HIV). Despite the prolonged survival due to treatment with a combined antiretroviral therapy (cART), HIV-infected patients are at an increased risk of chronic diseases and seem to develop many conditions typically associated with aging (15). Several reasons for the accelerated aging in HIV-infected patients were proposed (16, 17). Modern HIV treatment protocols often include more than one drug. Usually, two combinations are used, the first composed of two nucleoside reverse transcriptase inhibitors (NRTIs) and one nonnucleoside reverse transcriptase inhibitor (NNRTI), and the second that consists of two NRTIs and one protease inhibitor (PI). Growing evidence indicates that these drugs could have possible adverse effects on aging dynamics (18, 19).

Since the rate of aging is not the same in every part of the human body (1–3), combined changes in iron metabolism parameters (12) and telomere length (20) might be used as tools to monitor the whole-body aging in HIV-infected individuals. We opted to investigate the RTL as a representative biomarker of aging in proliferating cells (21), as well as the iron metabolism parameters as aging markers for both dividing and nondividing cells (14, 22). Combination of different, independent biomarkers of aging could provide a reliable approach in studying aging process at the level of the entire organism. Thus, we propose a novel, simple method that combines two types of relatively easily determinable parameters to predict the rate of aging. The aim of this cross-sectional study was to estimate the rate of biological aging in HIV patients of different chronological age under different therapy regimens.

## RESULTS

### Clinical data analyses.

The main clinical and epidemiological data regarding the HIV group are summarized in Table 1. The dominant HIV transmission pathway in older patients was through a heterosexual contact, while the younger patients were infected mostly during a nonprotected homosexual intercourse. All older HIV^+^ patients were on cART, as well as 47 younger HIV^+^ patients (85.5%), while 8 younger patients (14.5%) were naive. The older HIV-seropositive patients had received cART for a longer period of time compared to the younger patients (*P < *0.001). Also, blood levels of the following biochemical parameters were significantly lower in younger patients compared to older: the total cholesterol (*P = *0.017), low-density lipoproteins (*P = *0.001), and C-reactive protein (*P = *0.037).

**TABLE 1:** Clinical parameters of HIV-infected patients^*a*^

Variable	Mean ± SD or median (IQR)		*P*
	Younger patients (*n* = 55)	Older patients (*n* = 50)	
Age (yr)	29.2 ± 0.5	59.6 ± 1.1	**<0.001**
CD4 count (cells/μL)	461.9 ± 232.6 (10–984)	567.0 ± 317.7 (84−1,368)	0.131
pVL (copies/mL)	290.7 ± 112.7 (0–3,940.0)	235.0 ± 107.0 (0–2.8)	0.418
Therapy duration (mo)	28.9 ± 32.3 (0.8–128.8)	85.6 ± 61.1 (0.8–213.7)	**<0.001**
Transmission, *n* (%)			
Homosexual	40 (72.7)	13 (26.0)	**<0.001**
Heterosexual	6 (10.9)	**24 (48.0)**	**<0.001**
Blood transfusion	0 (0)	2 (4.0)	0.224
Vertical	0 (0)	0 (0)	
Unknown	9 (16.4)	11 (22.0)	0.313
Biochemical parameters			
BMI (kg/m^2^)	25.1 ± 2.9	25.9 ± 4.2	0.280
AST (U/L)	25.7 ± 9.8	25.5 ± 11.9	0.949
ALT (U/L)	38.7 ± 51.2	27.9 ± 11.4	0.220
ALP (U/L)	86.8 ± 30.1	91.5 ± 42.3	0.647
TC (mmol/L)	5.3 ± 1.3	6.1 ± 1.1	**0.017**
TG (mmol/L)	2.5 ± 2.3	2.6 ± 1.9	0.860
HDL (mmol/L)	1.0 ± 0.7	1.2 ± 0.6	0.511
LDL (mmol/L)	2.7 ± 0.8	3.9 ± 1.3	**0.001**
Glucose (mmol/L)	5.2 ± 0.4	5.9 ± 2.1	0.074
CRP (mg/L)	9.0 ± 13.2	20.4 ± 37.4	**0.037**

a Results are expressed as means ± the standard deviations or medians ± the interquartile range (IQR). The ranges for the CD4 count, pVL (in thousands), and therapy duration are given in parentheses. BMI, body mass index; AST, aspartate aminotransferase; ALT, alanine aminotransferase; TC, total cholesterol; TG, total triglycerides; HDL, high-density lipoprotein; LDL, low-density lipoprotein; CRP, C-reactive protein. Values in boldface are significant.

### Relative telomere length analyses.

The telomeres in younger patients were longer compared to older ones in both uninfected and HIV-infected patients. The patients with HIV infection tended to have longer telomeres compared to their uninfected counterparts, though the difference did not reach statistical significance (Table 2). In the uninfected group, telomeres of younger subjects were significantly longer compared to telomeres of older participants (*P = *0.026). However, in the HIV-infected group, telomeres of younger and older patients did not differ significantly (*P = *0.267) (Table 2).

**TABLE 2:** Comparison of RTL values between examined groups

HIV status/age	Age (yr)/HIV status	RTL^*a*^	*P* ^*b*^	*t* value	df
HIV status					
HIV^+^	<35	0.87 ± 0.37	0.216	−0.111	103
	>50	0.79 ± 0.32			
HIV^–^	<35	0.83 ± 0.36	**0.031**	−2.255	98
	>50	0.69 ± 0.24			
Age (yr)					
<35	HIV^+^	0.87 ± 0.37	0.512	0.476	103
	HIV^–^	0.83 ± 0.36			
>50	HIV^+^	0.79 ± 0.32	0.093	1.683	98
	HIV^–^	0.69 ± 0.24			

a Results are expressed as means ± the standard deviations. RTL, relative telomere length.

b Values in boldface are significant.

### Iron metabolism analyses.

**FIG 1:**
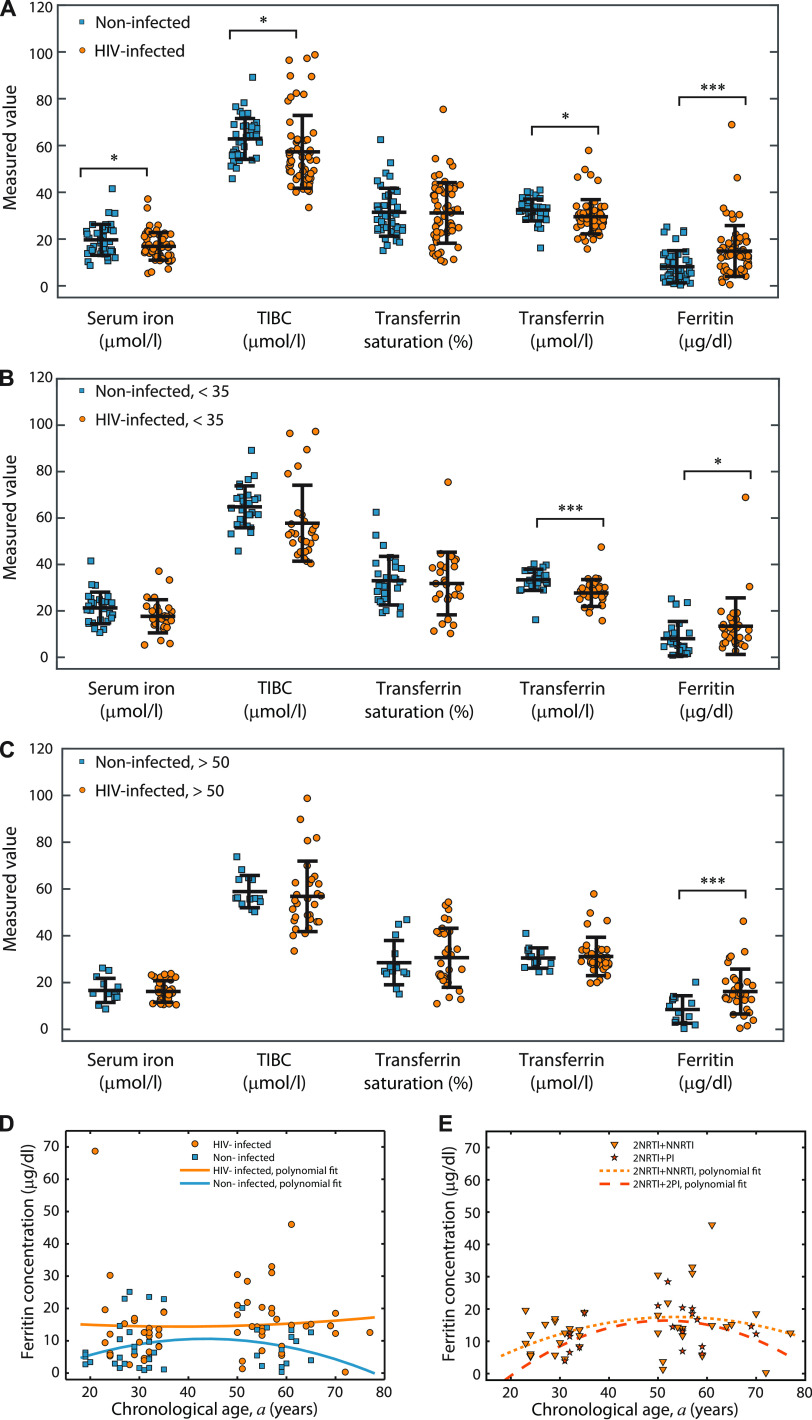
(A) Comparison of iron metabolism parameters levels in blood serum of uninfected and HIV-infected patients. (B) Comparison of iron metabolism parameters levels in blood serum of uninfected and HIV-infected patients younger than 35 years of age. (C) Comparison of iron metabolism parameters levels in blood serum of uninfected and HIV-infected patients older than 50 years of age. All data values, as well as the corresponding means ± standard deviations, are depicted for each parameter and patient group. Asterisks designate *P* values lower than 0.05 (*) and 0.001 (***). (D) Dependence of serum ferritin concentration on chronological age in uninfected and HIV-infected subjects. All data values as well as the second order polynomial fits are shown. (E) Dependence of serum ferritin concentration on chronological age in HIV-infected subjects treated with two different drug combinations. All data values, as well as the second-order polynomial fits, are shown.

In our studied population, we have found significantly higher values of serum iron (19.68 ± 6.62 μmol/L versus 16.87 ± 5.91 μmol/L), TIBC (62.82 ± 8.76 μmol/L versus 57.32 ± 15.57 μmol/L) and transferrin concentration (32.42 ± 4.66 μmol/L versus 29.54 ± 7.33 μmol/L) in the group of uninfected compared to HIV-infected patients. However, ferritin levels were significantly lower (*P < *0.001) in uninfected compared to HIV-infected subjects (6.50 ± 6.60 μg/L versus 14.87 ± 10.88 μg/L). The percentage of transferrin concentration did not differ between the examined groups (Fig. 1A).

In patients younger than 35 years of age, the transferrin concentration was significantly higher in uninfected than in the HIV-infected group (33.38 ± 4.57 μmol/L versus 27.69 ± 5.79 μmol/L) (Fig. 1B). The serum ferritin levels were significantly higher in the HIV-infected patients compared to their uninfected counterparts from younger (13.4 ± 12.19 μg/L versus 7.03 ± 7.23 μg/L) or older (16.16 ± 9.59 μg/L versus 4.68 ± 3.82 μg/L) group (Fig. 1B and C). We are not aware that anybody looked for distribution of these parameters in male HIV-infected patients younger than 35 and older than 50 years of age. In addition to these differences, the second order polynomial fits given in Fig. 1D reveal that in the uninfected group the dependence of the ferritin level on the chronological age followed the one already published (23), whereas the ferritin level in HIV-infected group was almost independent of the chronological age. The ferritin levels that correspond to the two considered drug therapy combinations did not exhibit a statistically significant difference. However, the corresponding second-order polynomial fits in Fig. 1E show that the curve shape of the 2NRTIs+NNRTI treatment was slightly closer to the one for the uninfected group, whereas the same can be said for the ferritin level of the 2NRTIs+PI drug combination.

### Relations between pVL, telomere length, and iron metabolism.

**FIG 2:**
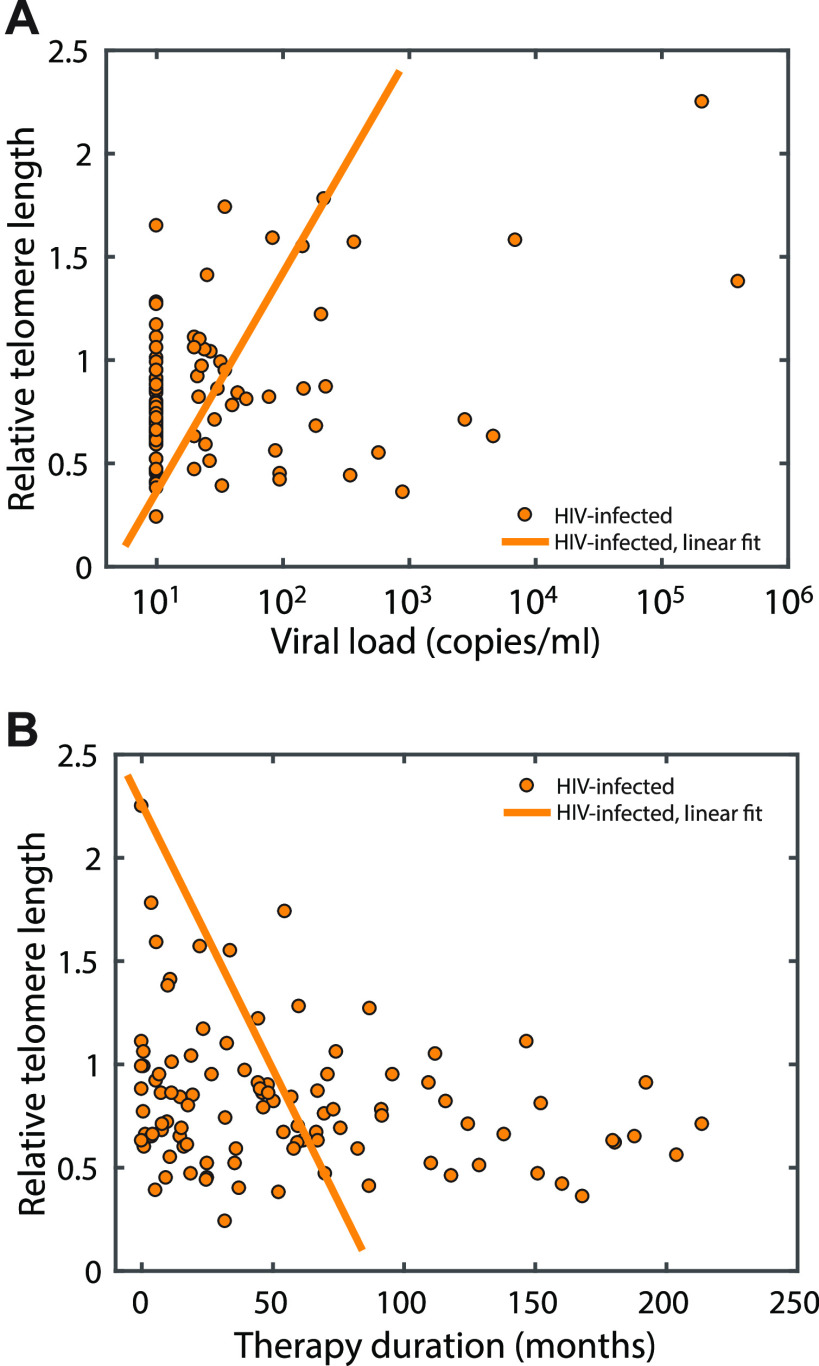
Scatter diagrams showing the relation of HIV pVL to RTL. To enable taking into account number of copies smaller than 20 it was assumed that they are all equal to 10 as a value in the middle of the range. (A) Relation between pVL and RTL; (B) relation between RTL and therapy duration.

A relation between pVL and telomere length was found (Fig. 2A). Higher plasma viral load was related with increase of telomere length. Also, a relation between therapy duration and RTL was present (Fig. 2B). Longer treatment with antiretroviral drugs was related with decrease of telomere length. There were no relations between HIV plasma viral load and iron metabolism parameters, nor between relative telomere length and the each of the iron metabolism parameters (data not shown).

### Biological aging.

**FIG 3:**
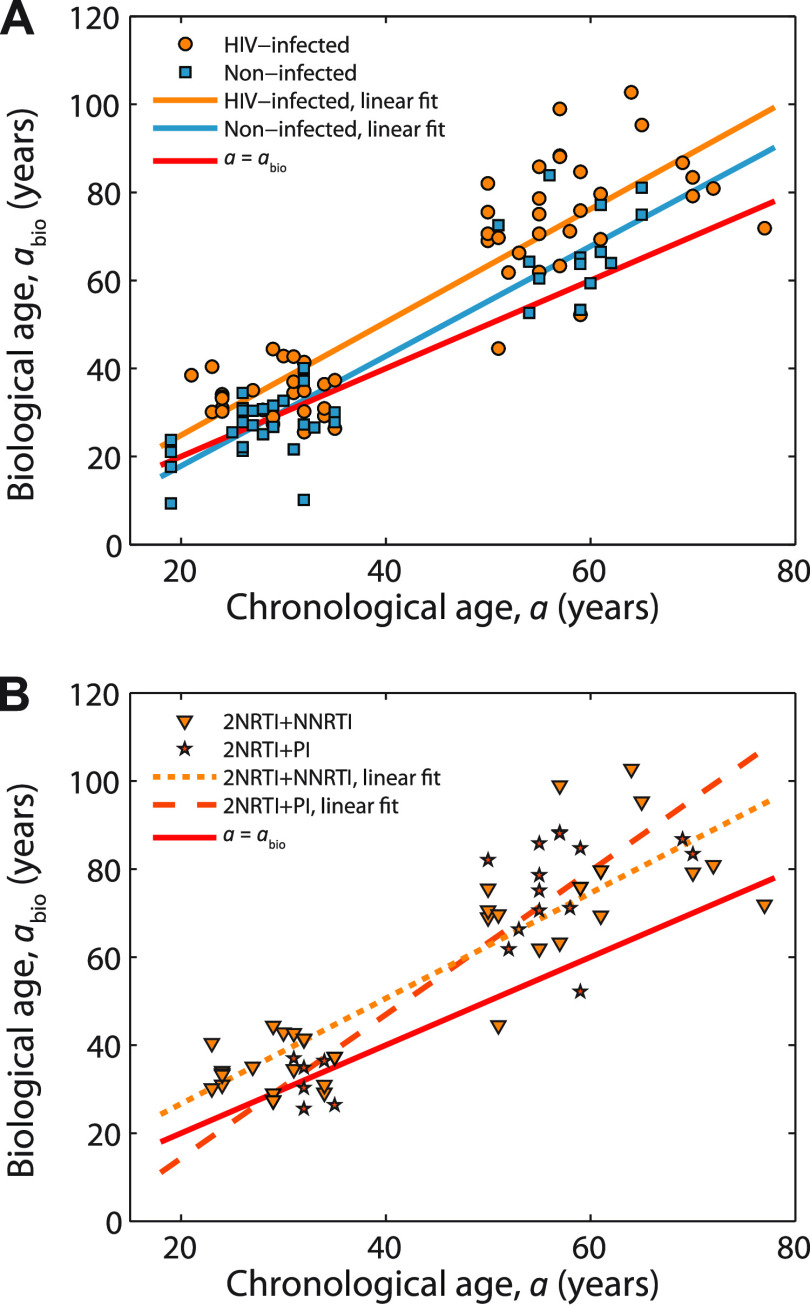
(A) Biological age dependence on chronological age. An approximate illustration of the biological age was calculated using equation 1 and Table 3. The subjects above the line *a* = *a*_bio_ are older biologically than chronologically, whereas those below this line are biologically younger than chronologically. (B) Biological age dependence on chronological age for treated patients. The patients treated with 2NRTI+NNRTI and patients that were subject to 2NRTI+PI treatment were considered separately. Note that the graph scaling is equivalent to the one in panel A, which enables comparison of the data presented in the two graphs.

The biological age as defined by the proposed formula (see equation 1) is given in Fig. 3A. The depicted results can be conceived as an illustration of the relationship between the biological and chronological age in the HIV-infected and uninfected groups. Also shown are linear fits to the referent population data, *a*_bio_ = *a*, as well as to the HIV-infected and uninfected groups. As expected, the HIV-infected group is biologically older than both the uninfected group and the referent population. Note that our uninfected group starts as being biologically younger than the referent population but then ages faster and after 29 years of chronological age become biologically older than the referent population.

The biological ages of the HIV-infected patients that were subjected to 2NRTIs+NNRTI and 2NRTIs+PI treatments were extracted (Fig. 3B), along with the corresponding linear fits.

The biological ages of patients treated with 2NRTIs+PI differ from those of uninfected volunteers more than is the case for HIV-infected patients treated with 2NRTIs+NNRTI (Fig. 3B). The 2NRTIs+PI treatment is favorable for younger patients because these patients are biologically younger than those treated with 2NRTIs+NNRTI. However, the 2NRTIs+PI treatment is associated with the steeper biological age dependence, as well as with biologically older patients in the group chronologically older than 50 years of age compared to the 2NRTIs+NNRTI treatment. Therefore, based on our biological age calculation, the 2NRTIs+NNRTI combination of antiretroviral drugs was overall better than the 2NRTIs+PI treatment.

## DISCUSSION

To the best of our knowledge, this is the first study in which telomere lengths and iron metabolism parameters in two different age groups of HIV-infected patients were compared in order to evaluate the impact of different therapy regimens on the aging process. The clinical characteristics of our study group were similar to other studies with a Caucasian population, except for the mode of HIV transition (Table 1). Namely, in our case the predominant HIV transmission pathway among older HIV-infected patients was unprotected heterosexual contact, as opposed to U.S. and European Caucasian populations, where older men who had sex with men (MSM) accounted for >85 and 40% of HIV-infected subjects, respectively (24, 25).

This study is focused on RTL and iron metabolism parameters that can be simply determined from a whole-blood sample and used for the relative assessment of a patient’s biological age. Whole blood is easily accessible and probably reflects the aging process in a body as a whole better than any other organ or tissue type. Determining the RTL values and iron content in each subpopulation of white blood cells would certainly provide more information about the aging process in each cell type in HIV-infected individuals, but it would also make the whole procedure more complex. Moreover, results collected from one cell subpopulation in our studied group may not fully represent biochemical processes of the whole body, even though some authors reported that the rate of telomere shortening in leukocytes is equivalent to those in adult solid tissues (11). The usual therapeutic approach in HIV-infected patients is based on the plasma viral load and CD4 count. Most of our patients reconstituted their immunity after the start of the treatment, and their viral loads dropped below detectability. Because of that, it was not feasible to use viral loads as a marker to compare drug efficiency in respect to the biological age. However, we have found a relation between the viral load and the telomere length (Fig. 2A) and consequently used the telomere length in our aging formula. In respect to the CD4 count, we decided to use whole blood as a better representative of not only the RTL but also of the amount of iron in the entire body. It has been shown that the amount of ferritin in blood correlates to the amount of iron in the whole body (26). Since the telomere length and ferritin determined from blood reflect their status in the whole body, we were able to generate the formula applicable to the entire organism. Involving additional biomarkers and analyzing different tissue types could contribute to a more precise assessment of biological aging rate. However, this study reports combination of two simply determined blood parameters as a novel approach for the analyses of the aging process in HIV-infected individuals.

The assessment of RTL changes in HIV-infected patients has certain limitations. For example, the dynamic of normal telomere shortening in the general population is not yet firmly defined (27). It has been shown that even newborns could have different telomere lengths, which could be affected by both intrinsic and extrinsic factors during a life period (28). The dynamics of changes in telomere length in HIV-infected patients should be evaluated from two or even more time points since the beginning of the infection and the start of the treatment are not necessarily the same in each patient. In the case of nondividing cells, mechanisms other than telomere shortening should be used for the explanation of cellular senescence. In spite of these limitations, we obtained the trend of longer telomeres in HIV-infected patients compared to the telomeres of healthy subjects (Table 2). It is possible that the HIV preserved or even upregulated the mechanisms of host telomere elongation and thus expanded the life cycle of the invaded host cell and prolonged the viral existence (29, 30). We propose that it is also feasible that the reverse transcriptase of HIV serves as a template for telomere elongation because of its structural (31) and functional similarity with human telomerase enzyme. We assumed that discrepancies between our study and other studies that reported telomere loss upon HIV infection could possibly arise due to different study designs, viral coinfections, and race, gender and lifestyle influences on telomere length. On the other hand, it is worth noting that antiretroviral therapy could accelerate telomere shortening. This could be due to the inhibition of human telomerase by NIRTs and NNIRTs or other side effects of PI therapy, which promote telomere loss (32, 33). In line with this is our finding that telomere length decreases with duration of therapy (Fig. 2B). In other words, therapy keeps virus under control but accelerates the biological aging process.

Most iron in an organism is bound to proteins, which serve as carrier or storage molecules. Upon absorption, iron is transported by transferrin to the target cells in which it is used for the synthesis of various biomolecules. The excess of iron is bound to the intracellular protein ferritin which represents the iron storage in the body (26). Our iron metabolism data are in accord with the findings of others and showed a pattern of anemia of chronic diseases (34). The novelty is that this pattern is maintained in groups of patients younger than 35 and those older than 50 years of age. We assumed that accumulation of body iron stores reflects the process of accelerated aging in the HIV-infected patients. On the other hand, some authors stressed the importance of iron in viral homeostasis and consequent decrease in hosts iron stores upon HIV infection (35). Rapid lowering of serum ferritin levels was reported after intermittent interruption of cART treatment within a Thai HIV-infected population (36). In order to replete iron stores in newly infected cells after cessation of therapy, a higher plasma viral load could decrease ferritin level in HIV-infected patients (36). Further studies are needed to clarify this issue.

The search for reliable indicators of biological age, rather than the chronological age, has been ongoing for some time. A number of different biomarkers have been proposed, with chronological age and the RTL being the most common ones (8, 10, 37). In addition to these two, we here used, for the first time, the iron parameters as biomarkers for the biological age in HIV-infected subjects. Compared to biomarkers used in other aging studies (9, 38, 39) that vary in nature and are more or less prone to relatively fast oscillations, our biomarkers are more stable and are physiological in nature. Based on different biomarkers, several attempts, using various statistical approaches (9, 40, 41), have been employed in order to develop the aging formula. Our formula for the biological age is specifically tailored to our needs; however, it is applicable to other studies and is particularly useful in situations where there is not a very large data set.

Two interesting results arose from our biological age formula. Namely, our uninfected patients were biologically older than the referent population (Fig. 3A). Older biological age could be possibly explained by different lifestyle events and conditions, and this assumption requires further sociological studies. It is significant that the life expectancy of the biologically older population of 72.9 years is shorter than that of the control population, 76.1 years (42). To the best of our knowledge, this is the first time that the human population life expectancy has been related to biological age. In line with these results, our HIV-infected patients were biologically older than the studied uninfected group (Fig. 3A). When taking different antiretroviral treatment modes into account, the biological aging became more complex. Namely, the 2NRTIs+PI therapy worked better in younger patients than the 2NRTIs+NNRTI therapy, but the 2NRTIs+PI therapy steeply accelerated the biological aging in the older patients (Fig. 3B). This could be due to chronic conditions that were commonly found in HIV^+^ patients receiving PIs (43, 44). On the other hand, the biological aging process in patients treated with 2NRTIs+NNRTI was more balanced than aging in patients on 2NRTIs+PI treatment (Fig. 3B). Overall, in respect to the biological aging process, the 2NRTIs+NNRTI combination of drugs was better than 2NRTIs+PI treatment (Fig. 3B).

The sample size limitation of our population was resolved by enlarging the pool of data with adding data from open access databases. Even though our study was based on data that corresponds to the state specific population, we have provided the formula adjustment that could translate our findings to various state censuses. An additional study limitation was a treatment with different combinations of antiretroviral drugs, which, due to their variety were classified into two main groups and thus potentially hid effects of the particular drug on biomarkers change. Moreover, our formula was intended to evaluate antiretroviral treatment efficiency and not the effects of the specific drugs, regarding biological aging.

The proposed formula for aging is introduced as an illustration of the biological age and can be treated as the first approximation in developing new strategies in the research of the aging process.

This study is the first, to the best of our knowledge, which has set the combination of two independent types of biomarkers in the assessment of biological aging and successfulness of drug treatment efficiency in HIV-infected patients. This approach could be used, as well, as a tool in the adjustment of individually tailored antiretroviral therapy. Furthermore, the same approach could be implemented in the evaluation of various physiological and pathological processes.

## MATERIALS AND METHODS

### Study design.

The present study included two groups of participants: the uninfected and the HIV-infected group. Both groups were subdivided into two subgroups: subjects younger than 35 years and subjects older than 50 years (45, 46). In each subgroup, the relative telomere length and five iron metabolism parameters—serum iron concentration, total iron binding capacity (TIBC), transferrin saturation, serum transferrin concentration, and ferritin concentration—were determined from whole blood samples. A mathematically derived formula, based on a combination of RTL and iron metabolism parameters, was used to calculate the rate of biological aging in HIV-infected patients on different therapy regimens.

### Subjects.

A total of 105 HIV-infected male patients treated at the HIV/AIDS Center of the Clinic for Infectious and Tropical Diseases, School of Medicine, University of Belgrade, Belgrade, Serbia, and 100 blood donor volunteers from the Blood Transfusion Institute of Serbia, Belgrade, Serbia, were enrolled in the study. All subjects were divided into two age groups: younger than 35 years and older than 50 years. Male patients only were recruited since adequate female groups could not be formed due to the small number of HIV-infected women in the studied population. HIV-infected patients included 97 patients on cART and eight untreated patients. Treated patients were divided into two groups: patients treated with 2NRTIs+NNRTI and patients treated with 2NRTIs+PI. Patients younger than 18 years and patients between 35 and 50 years were excluded from the study (45, 46). Additional exclusion criteria were coinfections with hepatitis B virus or hepatitis C virus, acute diseases, radiotherapy or cytotoxic drug therapy, and alcohol and/or narcotics abuse. The study protocol was explained in detail to the participants, and informed consent was obtained from each person. Due to the study design and availability of material, the data were gathered from 205 participants for the telomere length and 98 participants for the transferrin saturation. All other parameters were obtained from the groups whose sizes were between 205 and 98. All experiments were conducted in accordance with the Edinburgh Revision of Helsinki Declaration. The study was approved by the Ethical Committee of the School of Medicine, University of Belgrade (approval 29/XII-1).

### Sample collection and medical records.

Samples (10 mL) of whole venous blood were collected from each participant and used to perform standard biochemical analyses and to determine the RTL and the parameters of iron metabolism. The medical history, laboratory results, medication data, and other clinically relevant information were placed into specially designed study charts.

### Data collection.

In order to enlarge the pool and consequently improve the reliability and validity of our data, two additional publicly available U.S. population-based databases—The National Health and Nutrition Examination Survey (NHANES) and The Third National Health and Nutrition Examination Survey (NHANES III)—were used according to our strict inclusion and exclusion criteria. These surveys were conducted by the U.S. Centers for Disease Control and Prevention. Data from 2001–2002, 2003–2004, and 2005–2006 waves of the NHANES survey were used for obtaining RTL information and all iron parameter data except for ferritin data, whereas the NHANES III (1988 to 1994) database was used for obtaining ferritin values only (47).

### RTL measurement.

Relative telomere length (RTL) was determined from genomic DNA isolated from leukocytes of whole blood, as the representative of the processes that are ongoing in the entire body. RTL analyses were performed by quantitative PCR method, and each RTL was calculated using the ΔΔ*C_t_* method, as previously described (48).

### Determination of iron metabolism parameters.

Iron metabolism parameters, namely, serum iron, TIBC, transferrin saturation, and the concentrations of serum transferrin and ferritin, were determined by using commercially available kits according to the manufacturer’s recommendations (Biosystems, Barcelona, Spain).

### Plasma viral load and CD4 count.

Plasma viral load (pVL) was measured by a qRT-PCR using Ultrasensitive assay version 2.0 (Roche Molecular Systems, Branchburg, NJ), with a lower detection limit of 20 copies/mL (1.7 log_10_). The CD4 count was determined by flow cytometry using BD FACSCount CD4 reagent (Becton Dickinson Biosciences, San Jose, CA).

### Approximate quantitative biological aging illustration.

With the aim to offer a quantitative illustration of the biological aging component that can be related to the RTL and iron metabolism, a mathematically expressed parameter, denoted as the biological age, is introduced. This parameter and its relation to chronological age was used to approximately evaluate the biological aging in uninfected and HIV-infected patients, as well as to estimate biological aging in HIV-infected patients treated with two different combinations of antiretroviral drugs.

In order to enable appropriate use of qualitatively and quantitatively diverse variables in a single formula, as well as to avoid preferable or discriminating status of a variable caused merely by the unit that was used for its expression, all variables were normalized. The z-score normalization method that was used, which transforms a variable *X* into its normalized form, *X*_norm_, according to the formula:

$$X_{\text{norm}}=\frac{X-\mu }{\sigma }$$

where *X* is the value of a variable, whereas μ and σ are the mean value and standard deviation of the same variable in a population.

The central part of mathematical derivations is the formula that was intended to reflect the influence of the iron metabolism parameters, telomere length, and chronological age on the biological age. Consequently, the variables included in the expression for the biological age were the serum ferritin, free iron, TIBC, RTL, and chronological age. The transferrin saturation was not included in the biological age formula since it is a combined representation of the serum iron and TIBC, and the two were used in the biological age calculation as themselves. The serum transferrin concentration was not considered because it was estimated that its role as an aging indicator cannot be firmly justified, and it has a good correlation with TIBC as well (49). In addition to these parameters, for HIV-infected patients, it can be expected that the biological age is affected by the duration of illness. Consequently, the infection stadium was considered through the chronological age and life expectancy decrease for the treated HIV-infected population. The five parameters of interest were simultaneously available for 57 HIV-infected and 42 uninfected participants in our study. In order to provide desired statistical confidence of a formula for biological age, we also accessed publicly available data sets. In addition, we had to use two population data sets because a unique publicly available population data set that contained all our variables of interest was not available. Consequently, the correlation redundancy and principal-component analysis methods could not be used to determine biological age. Instead, the biological age, *a*_bio_, was defined as follows:

(1) $$\begin{array}{l} a_{\text{bio}}=k[\mathrm{sgn}\left(k_{\text{RTL}}\right){\mathrm{RTL}}_{\text{norm}}+\mathrm{sgn}\left(k_{\text{Fe}}\right){\mathrm{Fe}}_{\text{norm}}+\mathrm{sgn}\left(k_{\text{TIBC}}\right){\mathrm{TIBC}}_{\text{norm}}\\ +\mathrm{sgn}\left(k_{\text{F}}\right)F_{\text{norm}}+\mathrm{sgn}\left(k_{a}\right)a_{\text{norm}}]\;+C+a\Delta a_{\text{bioHIV–}}+\left(a-20\right)\Delta a_{\text{bioHIV}+} \end{array}$$

where RTL_norm_, Fe_norm_, TIBC_norm_, F_norm_, and *a*_norm_ are the normalized RTL, free serum iron, total iron binding capacity, serum ferritin, and chronological age, respectively, and *a* is the chronological age, whereas *k* and *C* are the scaling factor and constant that provide *a*_bio_ = *a* for the linear interpolation in the referent population.

The parameters μ and σ needed for normalization of variables, as well as *k*_RTL_, *k*_Fe_, *k*_TIBC_, *k*_F_, *k_a_*, *k*, and *C*, were determined from the NHANES and NHANES III databases. The parameters *k*_RTL_, *k*_Fe_, *k*_TIBC_, *k*_F_, and *k_a_* are defined as follows:

$$k_{X}=\frac{\Delta X_{\text{norm}}}{\Delta a}$$

They were calculated from the linear fits through the normalized population variables, namely, from the equation:

$$X_{\text{norm}}^{\text{pop}}=k_{x}a+n_{x}$$

If a particular parameter, *x*, is positive or negative, the corresponding sgn function, sgn(*x*), is equal to 1 or −1, respectively. The parameters *k* and *C* provide that the linear fit through the biological age calculated for the population data are equal to the chronological age and are:

$$k={\left(k_{\text{RTL}}\mathrm{sgn}(k_{\text{RTL}})+k_{\text{Fe}}\mathrm{sgn}(k_{\text{Fe}})+k_{\text{TIBC}}\mathrm{sgn}(k_{\text{TIBC}})+k_{\text{F}}\mathrm{sgn}(k_{\text{F}})+k_{a}\mathrm{sgn}(k_{a})\right)}^{-1}$$

$$C=-k\left(n_{\text{RTL}}\mathrm{sgn}(k_{\text{RTL}})+n_{\text{Fe}}\mathrm{sgn}(k_{\text{Fe}})+n_{\text{TIBC}}\mathrm{sgn}(k_{\text{TIBC}})+n_{\text{F}}\mathrm{sgn}(k_{\text{F}})+n_{a}\mathrm{sgn}(k_{a})\right)$$

Note that the contributions of the considered variables to the biological age are not multiplied by the weight factors. Instead, their changes with the chronological age in the population are normalized to 1 using the multiplication factor *k*. Consequently, the contribution of a considered variable to the biological age directly reflects its change with the chronological age in the population, *k|k_X_|*. Parameters *k_X_* and *k* are listed in Table 3.

**TABLE 3:** Population database parameters used as constants in biological age formula

Parameter	Group^*a*^	
	Younger participants (19 ≤ *a* ≤35)	Older participants (50 ≤ *a *≤ 77)
*μ_RTL_* ± *σ_RTL_*	1.1674 ± 0.2699	0.9310 ± 0.2211
*μ_Fe_* ± *σ_Fe_* (μg/dL)	106.701 ± 40.65551	92.84552 ± 34.4174
*μ_TIBC_* ± *σ_TIBC_* (μg/dL)	350.4698 ± 48.50848	340.4459 ± 51.6161
*μ_F_* ± *σ_F_* (μg/L)	141.099 ± 87.18172	195.7973 ± 171.4354
*μ_a_* ± *σ_a_* (yr)	27.4770 ± 4.7178	64.0432 ± 7.9415
*k_RTL_*	−0.0432	−0.0327
*k_Fe_*	−0.0270	−0.0086
*k_TIBC_*	−0.0183	−0.0096
*k_F_*	0.0608	0.0058
*k_a_*	0.2120	0.1259
*k*	2.7681	5.4744
*C*	27.4907	68.1219
Δ*a_bioHIV–_*	−0.044	−0.044
Δ*a_bioHIV+_* for HIV^–^	0	0
Δ*a_bioHIV+_* for HIV^+^	0.194	0.194

a Where applicable, the results are expressed as means ± the standard deviations.

Our subjects do not belong to the population that was used as the referent. In particular, our samples originate from the population whose life expectancy is *a*_life exp_ = 72.9 years (42), whereas the life expectancy of the population we used as the referent is *a*_ref life exp_ = 76.1 years (42). The parameter Δ*a*_bioHIV–_ = (*a*_life exp_ – *a*_ref life exp_)/*a*_life exp_ is used to adjust this discrepancy. Note that other numerous parameters that affect aging, but were not considered in our study, were to some extent taken into account with this adjustment. When applying the formula to subjects from any other population this adjustment should be set using the appropriate value of *a*_life exp_ or may be disregarded, depending on the objective of a study. The parameter Δ*a*_bioHIV+_ = (*a*_ref life exp_ – *a*_HIV+ life exp_)/(*a*_HIV+ life exp_ – 20) is used to take into account the difference in life expectancy for HIV– and treated HIV+ subjects, since life expectancy of HIV^+^ subjects whose treatment started when they were 20 is *a*_HIV+ life exp_ = 67 (50). This parameter was intended to take into account some of the illness-related parameters that affect aging, other than those considered in our study. As was the case with Δ*a*_bioHIV–_, the parameter Δ*a*_bioHIV+_ should be set to the appropriate value or disregarded when using the formula in some other study. There are indications that some of the variables considered here have different trends in the two age groups (23). For this reason, the two age groups were considered separately when calculating parameters in the biological age formula. The values of all the parameters are summarized in Table 3.

The parameters used in this study are not to be considered as a complete, overall combination of biological age indicators that are applicable to all systems but rather as an illustrative, early approach that may lead to further improvements.

### Statistical analyses.

All statistical analyses were performed using the SPSS 20.0 statistical package (SPSS, Chicago, IL). The normality of distribution for all tested variables was assessed by the Kolmogorov-Smirnov test. The differences between the studied groups with respect to the blood serum levels of iron metabolism parameters and relative telomere length values were determined with an independent samples *t* test or the Mann-Whitney U test, as appropriate. The results are presented as means ± the standard deviations or as medians ± the interquartile range. Differences were considered significant if a *P* value was <0.05. To enable taking into account viral copies lower than 20, we used value 10 representing the mean of undetectable range. The linear and second order polynomial fits were used to assess relations between various considered variables.
